# Validation of a physiological model for controlling a thermal head simulator

**DOI:** 10.1186/2046-7648-4-S1-A73

**Published:** 2015-09-14

**Authors:** Natividad Martínez, Agnes Psikuta, Simon Annaheim, José Miguel Corberán, René Michel Rossi

**Affiliations:** 1 Laboratory for Protection and Physiology, Empa, Switzerland; 2 Department of Applied Thermodynamics, Polytechnic University of Valencia, Spain; 3 HOPE-Helmet OPtimization in Europe, COST Action TU1101 WG4

## Introduction

The head plays an important role in human thermoregulation. Helmets typically provide additional thermal insulation that impairs heat dissipation, reducing comfort and user acceptance [1].

Thermal head manikins allow analysis of the local heat transfer properties of headgear, but they usually do not provide information about human thermal response. Physiological models allow simulation of local physiological reactions and the thermal effect at the skin surface. However, they cannot account for complex heat and mass exchange processes at the skin surface when protective equipment is worn.

We aim at controlling a thermal head manikin with a physiological model to develop a novel advanced method for headgear evaluation. This work presents the validation of the aforementioned physiological model by Fiala [2,4] (FPC model version 5.3, Ergonsim, Germany) for prediction of global and local temperatures at the head-site, specially needed for the coupling with body part manikins, and is going to be used as a reference for validation of the coupled thermal head simulator.

## Methods

A total of 41 exposures representing a wide range of environmental conditions, activity and clothing accompanied by descriptions of experimental protocol have been simulated using FPC model. This database contained data for rectal temperature, mean and local skin temperatures collected at different body locations. The precision of the FPC predictions was assessed by root-mean square deviation (rmsd).

## Results

**Figure 1:**
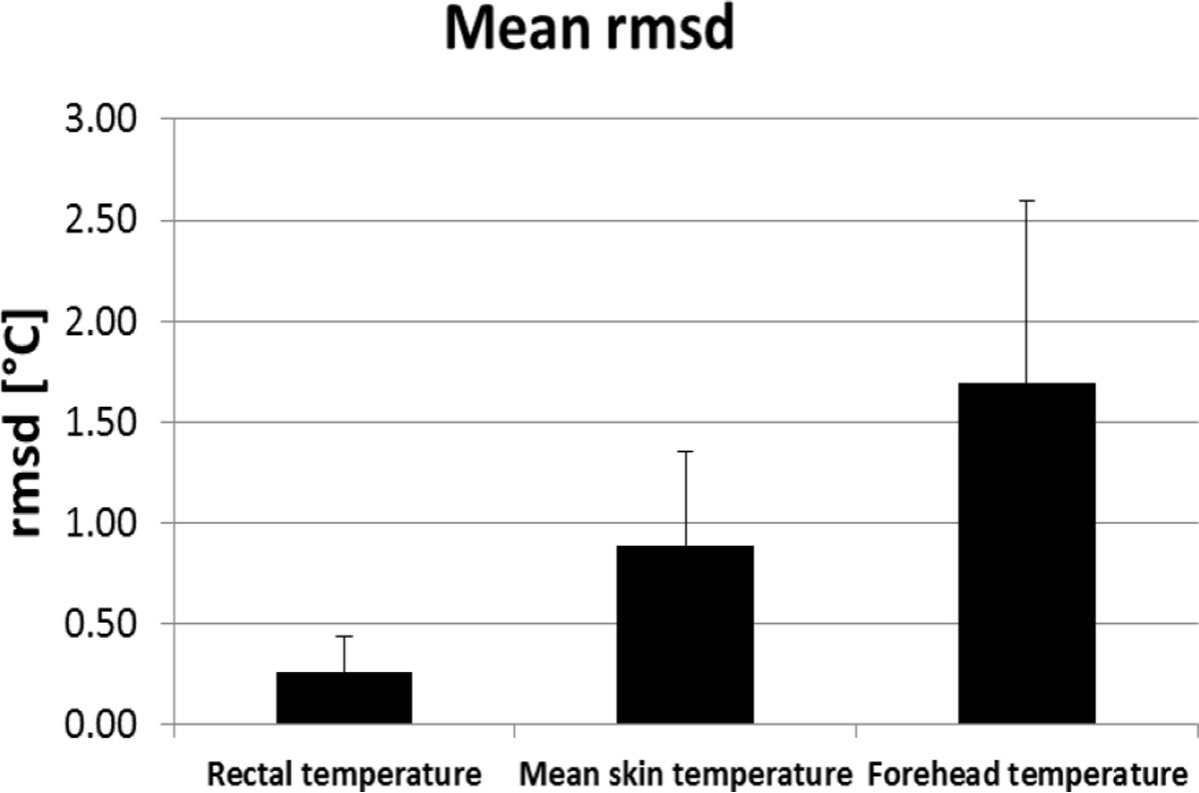
**Mean rmsd values for 41 exposures**.

Figure 1 shows mean precision values obtained for rectal, mean and local skin temperature at forehead.

## Discussion

The physiological model performed acceptably well when predicting overall physiological response but showed a greater discrepancy with experimental data for forehead skin temperature. This could be due to the presence of some additional insulation due to hair, displacement of the headgear over the sensors or allowance of moving the head during the data recording (e.g. avoiding direct exposure to wind).

## Conclusion

The validation of the physiological model provides a reference for assessing performance of the coupled thermal head simulator. The thermal head simulator will predict human thermophysiological response in different cases of use of headgear.
